# *bis*-Nitrile and *bis*-Dialkylcyanamide Platinum(II) Complexes as Efficient Catalysts for Hydrosilylation Cross-Linking of Siloxane Polymers

**DOI:** 10.3390/molecules21030311

**Published:** 2016-03-05

**Authors:** Regina M. Islamova, Mikhail V. Dobrynin, Daniil M. Ivanov, Andrey V. Vlasov, Elena V. Kaganova, Galina V. Grigoryan, Vadim Yu. Kukushkin

**Affiliations:** 1 Institute of Chemistry, Saint Petersburg State University, 7/9 Universitetskaya Nab., 199034 Saint Petersburg, Russia; mdobrunin42@yandex.ru (M.V.D.); dan1510@rambler.ru (D.M.I.); andreivl25@rambler.ru (A.V.V.); 2 Federal State Unitary Enterprise S.V. Lebedev Research Institute for Synthetic Rubber, Gapsal’skaya, 1, 198035 Saint Petersburg, Russia; vniisk-lab7@yandex.ru (E.V.K.); office@fgupniisk.ru (G.V.G.)

**Keywords:** platinum nitrile complexes, hydrosilylation, cross-linking, catalysis, silicon rubber

## Abstract

*cis*- and *trans*-Isomers of the platinum(II) nitrile complexes [PtCl_2_(NCR)_2_] (R = NMe_2_, N(C_5_H_10_), Ph, CH_2_Ph) were examined as catalysts for hydrosilylation cross-linking of vinyl-terminated polydimethylsiloxane and trimethylsilyl-terminated poly(dimethylsiloxane-*co*-ethylhydrosiloxane) producing high quality silicone rubbers. Among the tested platinum species the *cis*-complexes are much more active catalysts than their *trans*-congeners and for all studied platinum complexes *cis*-[PtCl_2_(NCCH_2_Ph)_2_] exhibits the best catalytic activity (room temperature, *c* = 1.0 × 10^−4^ mol/L, τ_pot-life_ 60 min, τ_curing_ 6 h). Although *cis*-[PtCl_2_(NCCH_2_Ph)_2_] is less active than the widely used Karstedt’s catalyst, its application for the cross-linking can be performed not only at room temperature (*c* = 1.0 × 10^−4^ mol/L), but also, more efficiently, at 80 °C (*c* = 1.0 × 10^−4^–1.0 × 10^−5^ mol/L) and it prevents adherence of the formed silicone rubbers to equipment. The usage of the *cis*- and *trans*-[PtCl_2_(NCR)_2_] complexes as the hydrosilylation catalysts do not require any inhibitors and, moreover, the complexes and their mixtures with vinyl- and trimethylsilyl terminated polysiloxanes are shelf-stable in air. Tested catalysts do not form colloid platinum particles after the cross-linking.

## 1. Introduction

Catalytic hydrosilylation, *viz.* catalytic addition of silicon hydrides to multiple bonds, is among the most important reactions in the silicone industry, in particular, for hydrosilylation cross-linking of multifunctional silicone hydride polymers with multi-vinyl functional silicon polymers giving broad spectrum of the silicone compositions of superior practical importance [1,2,3,4,5,6,7,8,9,10,11,12,13,14,15,16,17,18,19,20,21,22,23,24,25,26,27,28]. It is noteworthy that reported data on metal-catalyzed hydrosilylation cross-linking of vinyl *poly*siloxanes is substantially less abundant than those for *monomeric* dienes.

Hydrosilylation is in many respects relevant to hydrogenation, and it is not surprising that catalytic systems applied for hydrogenation are tested and successfully used for hydrosilylation reactions. Taking the analogy between two reactions into account, Pt- and Rh-based systems applied for various hydrogenations are usually considered as the most promising for hydrosilylation. Although a number of new catalysts for hydrosilylation of vinyl polysiloxanes have emerged in the past two decades [7,16,19,21,25], Karstedt’s complex (or its modified analogs) still remains the most widely used catalyst for cross-linking [7,21]. Its main disadvantage is hyperactivity that makes the cross-linking unselective even at room temperature (RT) and requires application of an inhibitor that, in many instances, impairs the strength characteristics of formed silicone formulations [29]. Moreover their use, in some circumstances, can be complicated by the colloidal platinum particles that form after reaction, which scatter light and which can render the silicone yellow.

Bearing in mind a great applied significance of hydrosilylation cross-linking for creation of silicone compositions, development of new efficient catalysts for these processes is always a challenging task. Besides general requirements to novel catalytic systems such as higher selectivity, tunable activity, and stability of the catalyst upon storage, the search for the new catalytic systems for the cross-linking includes design of catalysts that are shelf-stable at ambient temperature, but start to act at higher temperatures, as any premature curing would cause silicone polymers to adhere to equipment parts (e.g., rollers) requiring frequent shutdown for cleaning.

In the framework of our projects aimed, on the one hand, toward verification of catalytic properties of metal species in organic transformations (for recent works and for major reviews from our group on this topic, see Refs. [30,31,32,33,34]) On the other hand, on reactivity of platinum nitrile complexes (for recent work from our group, see Refs. [31]), we focused our attention on the possibility of applying platinum(II) nitrile species in hydrosilylation cross-linking of vinyl polysiloxanes. The starting point for this work was an intriguing study by Brook and coworkers [35], where the [PtCl_2_(NCC_6_H_4_C_6_H_4_OC_3_H_6_SiR_3_)_2_] complexes were employed as good catalysts for hydrosilylation of *monomeric* dienes. Although the isomeric configuration of these complexes was not elucidated, it was most likely *trans* as the synthetic scheme included prolonged heating (20 h) at elevated temperature (110 °C), *i.e.*, harsh conditions typically yielding *trans*-configured nitrile Pt^II^ species [36,37,38], especially those featuring sterically encumbered ligands.

Our basic idea included application as catalysts for hydrosilylation cross-linking of vinyl polysiloxanes of nitrile platinum(II) species in *cis*- and *trans*-isomeric forms and bearing both conventional nitriles RCN (R = Alk, Ar) and the so-called push-pull nitriles [33] such as dialkylcyanamides (R = NAlk_2_). We found that the *cis*- and *trans*-isomeric platinum(II) complexes, *viz.* [PtCl_2_(RCN)_2_] (R = NMe_2_, N(C_5_H_10_), Ph, CH_2_Ph), behave as efficient and practical catalysts for silicone polymers cross-linking giving high-quality silicone rubbers. Accordingly, we aimed to (i) search for the most efficient catalytic system, verify its structure–reactivity relationships, and compare the catalytic activity of our complexes to that of Karstedt’s complex; (ii) optimize catalytic cross-linking of vinyl-terminated polydimethylsiloxane (PDMS) and trimethylsilyl-terminated poly(dimethylsiloxane-*co*-ethylhydrosiloxane) (EHDMS), and (iii) examine the properties of the obtained silicone rubbers.

## 2. Results and Discussion

### 2.1. Monitoring of the Cross-Linking and Catalytic Studies

**Scheme 1:**
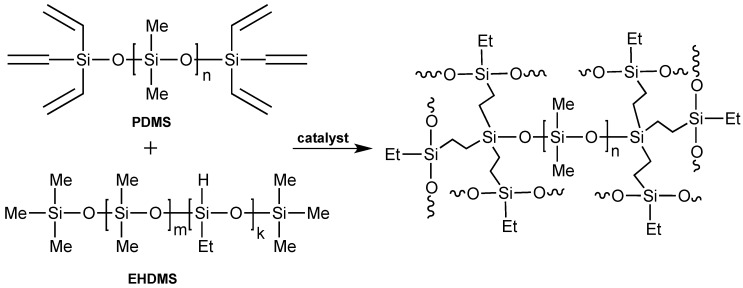
Platinum-catalyzed hydrosilylation cross-linking of siloxane polymers.

**Figure 1:**
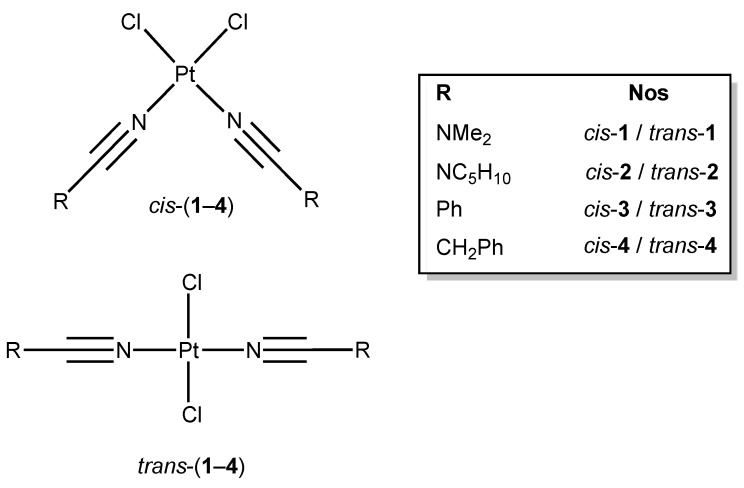
Platinum complexes employed as hydrosilylation cross-linking catalysts.

Platinum-catalyzed hydrosilylation cross-linking was studied for siloxane polymers PDMS and EHDMS (Scheme 1) at RT and at 80 °C and catalyst concentration range of 10^−3^−10^−5^ mol/L. The isomeric platinum(II) complexes featuring benzyl- and phenylcyanides and also dialkylcyanamides *cis*-(**1**−**4**) and *trans*-(**1**−**4**) (Figure 1) were examined as catalysts.

**Figure 2:**
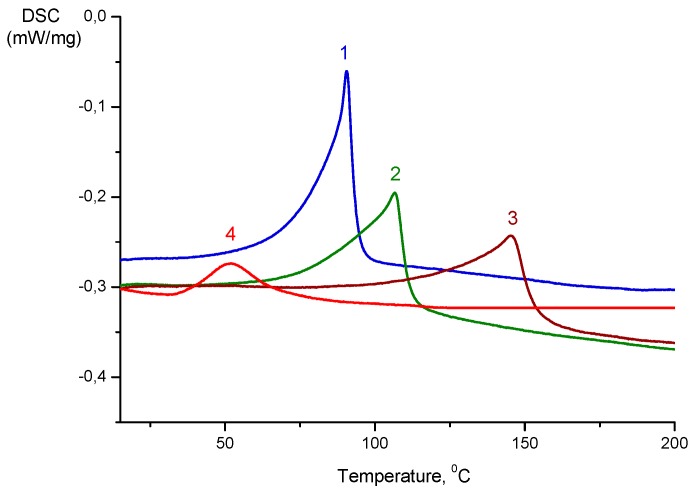
DSC curves of the curing catalyzed by: *cis*-**4** (1.0 × 10^−4^ mol/L) (1); *cis*-**4** (5.0 × 10^−5^ mol/L) (2); *cis*-**4** (1.0 × 10^−5^ mol/L) (3); Karstedt’s catalyst (1.0 × 10^−5^ mol/L) (4).

The cross-linking of PDMS and EHDMS catalyzed by any one of *cis*-(**1**−**4**) and *trans*-(**1**−**4**) was monitored by DSC and IR methods. The pot-life (τ_pot-life_) and curing time (τ_curing_) determined under different experimental conditions are given in Table 1. Table 1 provides compilation of these parameters obtained under analogous conditions with variation of either concentration or temperature. All DSC curves exhibit exothermic peaks (Figure 2) indicating the reaction and/or phase transition. Temperatures onset (T_onset_) and peak (T_peak_) (Table 1) are related to the beginning and the completion of the Pt^II^-catalyzed cross-linking upon heating. In addition, EHDMS consumption was monitored by the IR method indicating gradual decrease of the ν(Si−H) band (2152 cm^−1^) followed by stabilization of its intensity. This stabilization corresponds well to τ_curing_ (Table 1, Figures S1 and S2).

As can be inferred from inspection of the data compiled in Table 1, the most efficient catalyst among the tested platinum species is *cis*-**4** (R = CH_2_Ph). When this complex is used as the catalyst at 1.0 × 10^−3^ mol/L, τ_pot-life_ and τ_curing_ are 20 min and 3 h, correspondingly, whereas at 1.0 × 10^−5^ mol/L τ_curing_ is 30 day. The optimized conditions for preparation of silicone rubbers is RT and *c* = 1.0 × 10^−4^ mol/**L** with *τ*_pot-life_ 60 min and τ_curing_ 6 h. Complexes *cis*-**1**–**3** as catalysts for the cross-linking at RT should be used at higher concentrations (1.0 × 10^−3^ mol/L) to reach reasonable values of τ_pot-life_ and τ_curing_.

In contrast to Karsted’s catalyst that is quite reactive at RT, and can be used upon heating only combined with inhibitors [7,21], any one of *cis*-(**1**–**4**) efficiently acts at 80 °C and does not require other additives. When 1.0 × 10^−3^ mol/L of *cis*-(**1**−**3**) is used at 80 °C, the τ_curing_ does not exceed 10−20 min, and, in the case of most efficient *cis*-**4**, the cross-linking takes 1 h at the catalyst concentration 5.0 × 10^−5^ mol/L.

Complexes *cis*-**1**–**4** during the cross-linking release higher energy (ΔH) than Karstedt’ catalyst and this collaterally implies that the number of vinyl groups involved in the cross-linking is higher upon usage of the nitrile platinum catalysts (Table 1). However, the difference should not be large as the swelling experiments (see later) indicated rather comparable soluble fraction and polymer volume fraction values for both rubbers.

**Figure 3:**
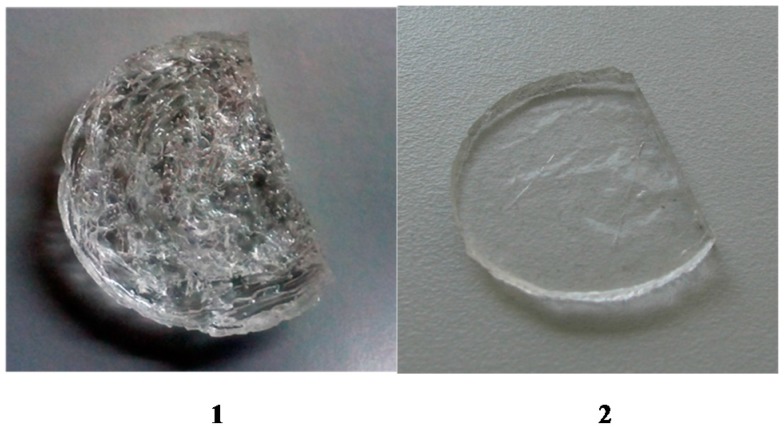
Photographs of the PDMS−EHDMS rubbers obtained in the presence of Karstedt’s catalyst (1.0 × 10^−5^ mol/L) (**1**) and *cis*-**4** (5.0 × 10^−5^ mol/L) (**2**) at RT, τ_curing_ is 6 h and 36 h, respectively.

The catalytic behavior of *cis*-**4** was compared with that of Karstedt’s catalyst (Table 1). The usage of the latter has certain disadvantages. Firstly, it must be used and stored in an inert gas atmosphere (solution in xylene, Sigma-Aldrich, St. Louis, MO, USA), without moisture, oxygen, and light. Secondly, Karstedt’s catalyst has hyperactivity that is accompanied with premature curing of vinyl-containing polysiloxanes by hydrosilylation already upon mixing the components that reflects in low values of τ_pot-life_ and τ_curing_. The fast cross-linking leads to the worsening in the quality of the resulting polysiloxane rubber, namely, to the formation of bubbles and other structural defects (Figure 3).

Thirdly, the reaction mixture persistence to early curing, as a rule, is attained by the introducing inhibitors such as, for instance, maleates, fumarates, nitriles, *etc.*, retarding the curing [5,7,21]. However, the inhibitor introduction complicates the catalytic system. In addition, the possibilities of inhibitors are limited because, according to Ref. [29], in some cases, the strengthening properties of the obtained silicone resins are worsened. Catalyst *cis*-**4** is free from these disadvantages as it does not require inert atmosphere and the usage of inhibitors, and it is shelf-stable.

We found that the platinum(II) nitrile complexes of the *trans*-configuration are much less active than their *cis* congeners (Table 1). Using *trans*-(**1**–**4**) at RT, the time of complete curing varies from 1 day to 1 month. *Trans*-nitrile complexes **1**–**4** are most effectively operate at 80 °C (Table 1, Figure S3). The curing time of PDMS and EHDMS in the presence of *trans*-(**1**–**4**) is 120, 80, 60, and 15 min, respectively. All studied *cis*-(**1**–**4**) and *trans*-(**1**–**4**) species are shelf-stable preserving their catalytic activity in polysiloxane solution in air for at least 30 day as confirmed by the DSC method (Figure S4).

The hydrosilylation efficiency in siloxane system in the presence of *cis*-(**1**–**4**) and *trans*-(**1**–**4**) depends on both the isomeric structure and the nature of substituents in the nitrile ligands. First of all, the catalytic activity greatly depends on the geometric structure of the complexes and, for complexes of the same composition, the *cis*-isomer is always more catalytically active that the *trans*-isomer. Among the studied *cis*-nitrile complexes, the catalytic activity is decreased along the following series: *cis*-**4** (PhCH_2_) > *cis*-**3** (Ph) > *cis*-**2** (NC_5_H_10_) ≈ *cis*-**1** (NMe_2_), where *cis*-**4** exhibits substantial activity, whereas all remaining *cis*-species possess lower activities. It is noteworthy that the indicated consequence is approximate insofar as our bulk experiments do not take into account a possibility of the well-known *cis*-to-*trans* isomerization of the nitrile platinum(II) species [36,37,38].

**Scheme 2:**
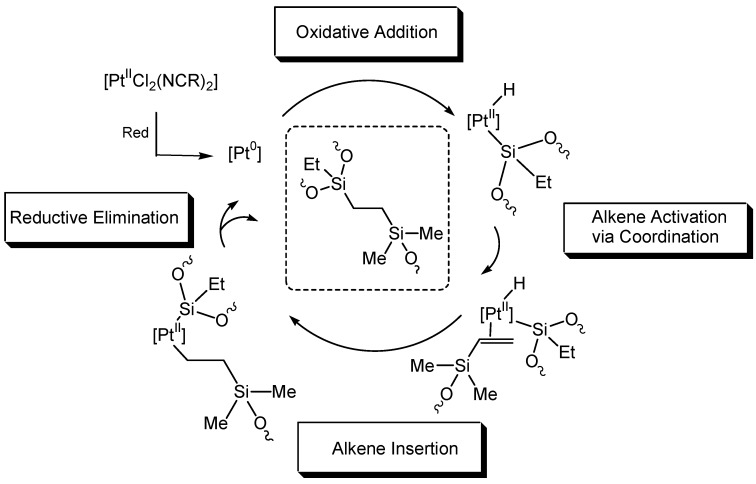
Plausible mechanism of the cross-linking.

Platinum-catalyzed hydrosilylation of olefins is commonly described by the Chalk–Harrod mechanism or its modified version [7,21]. We believe that in the studied system, platinum(II) centers of the nitrile complexes are reduced by either silane- or vinyl groups to give platinum(0) species (Scheme 2) that are involved in oxidative addition with EHDMS. Vinyl groups of PDMS coordinate to the formed platinum(II) centers, undergo activation followed by their insertion into the Si–H bond. Reductive elimination with regeneration of the catalytic species terminates the process. Unfortunately, all our attempts to detect the catalytic species by high resolution electrospray ionization mass spectrometry (HRESIMS) failed due to substantial overlap of peaks from platinum species and the polymeric system.

### 2.2. Properties of the Silicone Rubbers

In contrast to rubbers obtained with Karstedt’s catalyst, our PDMS-EHDMS silicone formulations bear no bubbles or other structural defects (Figure 3), and their surface is homogeneous and transparent. They can be stored in the light and in air for at least one year without visible changes. The rubbers were analyzed by DSC and TG methods, and we also performed swelling measurements. In addition, their dynamic characteristics and elastic properties were studied by the dynamic mechanical analysis DMA method and by conducting tensile tests, respectively. All our results are consistently disclosed in paragraphs that follow.

Silicone rubber obtained with *cis*-**4** or with Karstedt’s catalyst are characterized by approximately equal melting (*ca.* −40 °C) and crystallization (*ca.* −70 °C) temperatures, and the melting (−25 J/g) and crystallization (−22 J/g) enthalpies (Figure S5). These results indicate that the nitrile platinum catalysts do not affect the phase transitions upon both heating and cooling of the obtained siloxane rubbers.

In the TG curves, the temperature of the initial weight loss of the silicone rubber obtained with *cis*-**4** under argon and in air increases by (80−90) and (30−40) °C, respectively, to these TG data obtained for Karstedt’s catalyst (Figures S6 and S7). Final residues of the silicone rubbers obtained with *cis*-**4** and Karstedt’s catalyst (both at 1.0 × 10^−5^ mol/L) remaining after heating the samples up to 800 °C are *ca.* 80% and 40% [under argon] or *ca.* 70% and 55% [in air], respectively. These TG data indicate that the usage of *cis*-**4** improves thermal stability and flame (fire) retardancy of rubbers compared with those obtained with Karstedt’s catalyst. Weight loss shifts to a higher temperature both in argon and in air. The mechanism of thermal degradation of silicone rubbers was previously described [39,40].

Swelling measurements give information about soluble fraction (w_sol_) and polymer volume fraction (υ). The optimal networks formed at smaller soluble fraction and minimum swelling in good solvent (toluene is a good solvent for PDMS). As expected, there is a close correlation between the values of w_sol_ and υ. The smaller w_sol_ and the greater υ are for each series of networks [41,42,43]: w_sol_ are 5.8% and 5.1% and υ are 0.21 and 0.22 in the presence of *cis*-**4** (1.0 × 10^−5^ mol/L) and Karstedt’s catalyst (1.0 × 10^−5^ mol/L), respectively. The difference in values of the swelling experiments in the presence of *cis*-**4** and Karstedt’s catalyst is not substantial. More complete information about cross-linking process gives ΔH of the process (see above).

Inspection of the dynamic characteristics of the siloxane rubbers obtained with *cis*-**4** (1.0 × 10^−5^ mol/L) indicates that the temperature is lowered, a sharp increase in the elastic modulus E', and the passage through the highs tangent of loss angle, indicating crystallization study of siloxane rubbers [44]. Upon further cooling, the amorphous phase becomes vitrified state, but difficult fixation of this transition high crystallinity of the samples. When heated, the process is reversed. Noteworthy that the highest value of the elastic modulus are characteristic of the sample that was obtained with *cis*-**4** (E′ 2.5 MPa at RT) and the lowest for the Karstedt’s catalyst (E′ 0.4 MPa at RT). We assume that, in the presence of *cis*-**4,** more cross-linked sample bearing less network defects is obtained.

Elastic properties of our silicon rubbers have a relatively high elongation at break (L) and tensile strength (σ), but relatively little residual elongation (Δl) compared to the rubbers obtained with Karstedt’s catalyst. The σ values are 0.25 and 0.22 MPa, L are 235% and 170%, Δl are 5% and 10% in the presence of *cis*-**4** (1.0 × 10^−5^ mol/L) and Karstedt’s catalyst (1.0 × 10^−5^ mol/L), respectively.

## 3. Experimental Section

### 3.1. Materials and Instrumentation

Solvents were obtained from commercial sources and used as received. Complexes *cis*- and *trans*-(**1**–**4**) were prepared as previously reported [45,46,47]. The chemicals for evaluation of catalytic activity of **1**–**4** were obtained from the following sources, *viz.* 0.1 M Karstedt’s catalyst in vinyl terminated polydimethylsiloxane (Aldrich); trivinyl terminated polydimethylsiloxane (PDMS; the weight average molecular weight M_w_ = 80000, the number average molecular weight M_n_ = 33800, 0.5 wt % of CH=CH_2_) and trimethylsilyl terminated poly(dimethylsiloxane-*co*-ethylhydrosiloxane) (EHDMS; the weight average molecular weight M_w_ = 8150, the number average molecular weight M_n_ = 4600, 0.7 wt % of Si–H) from FSUE NIISK, Saint Petersburg, Russian Federation.

The progress of the cross-linking was monitored by (i) DSC method using a NETZSCH DSC 204F1 Phoenix instrument (Naperville, IL, USA). DSC conditions are as the following: cooling from 25 to −10 °C, heating from −10 to 220 °C, and then cooling from 250 to 25 °C (10 °C/min); all components were mixed before DSC studies and DSC experiments were conducted twice for each test; (ii) IR method using Shimadzu FTIR-8400S instrument (Kyoto, Japan) at RT.

DSC of cured materials was performed at a NETZSCH DSC 204F1 Phoenix instrument: cooling from 25 to −100 °C, heating from −100 to 50 °C at 5 °C/min under argon and in air. DSC experiments were repeated twice for each test. TG measurements were performed on a NETZSCH TG 209F1 Libra instrument (Burlington, MA, USA); 2 mg of sample was taken in a platinum pan that was then heated from RT to 900 °C at 10 °C/min under a flow of argon (50 mL/min) or in air.

Dynamical mechanical analysis was performed on NETZSCH DMA 242C. Sample characteristics: length 5 mm, width 12 mm, height 2 mm. DMA conditions: cooling from 25 to −150 °C, heating from −150 to 50 °C at 5 °C/min and 1 Hz in air. Tensile tests were performed on Zwick Proline Z005 (Ulm, Germany) tensile testing machine. Tensile tests followed ISO 34, these experiments were carried out five times for each test.

### 3.2. Catalytic Studies

#### 3.2.1. Catalytic Tests in Polymeric Systems

The cross-linking was studied in a system consisting of PDMS and EHDMS as cross-linking partners and a platinum catalyst, *viz.* any of one *cis*- and *trans*-(1−4) or Karstedt’s catalyst taken for comparison, at 23 or at 80 °C. A ratio Si‒H:CH_2_=CH_2_ is 3:1. The standard formulation had [part A]:[part B] = 1:1 ratio; concentration of 1−4 was 1.0 × 10^−3^−1.0 × 10^−5^ mol/L. [Part A]: calculated amounts of 1−4 (diluted in CH_2_Cl_2_) were added to PDMS, the mixture was carefully stirred and dried under vacuum at 30 °C overnight to remove the solvent. Karsted’s catalyst dissolved in vinyl-terminated polydimethylsiloxane was added to PDMS without drying in a vacuum. [Part B]: EHDMS (10 g) was added to PDMS (50 g) and this mixture was carefully stirred for homogenization ([−CH=CH_2_]/[≡Si−H] = 1:3).

#### 3.2.2. General Procedure of Curing Time and Pot-Life Determination

The cross-linking process of the polysiloxanes mixture was performed in an aluminum vessel. The pot-life time was measured from the point of mixing equal amounts of Parts A and B before the viscosity of the initial mixture increases to an unusable level at RT [30]. Typically, the pot-life is defined as the time when viscosity of catalyzed mixture is doubled. The curing time was measured from the point of mixing equal amounts of Parts A and B at RT or from the moment of placing the aluminum vessel with equal amounts of Parts A and B mixture to a desiccator at 80 °C until liquid reaction mixture getting dry cured material.

#### 3.2.3. Swelling Measurements

A typical sample of obtained PDMS‒EHDMS with platinum catalyst rubber (0.1 g, cylindrical disk of 10 mm diameter and 1 mm thickness) was weighted for evaluating the initial dry weight (m_unex_), swollen by Soxhlet extractor for one day, and then plunged in toluene (50 mL) in a sealed bottle for 2 h in the dark. The sample was extracted, gently wiped to remove liquid solvent present on the sample surface and immediately weighted (m_s_). The sample was then dried overnight at RT and then 2 h at 60 °C under vacuum and reweighted (m_ex_). Soluble fraction (w_sol_) and volume fraction of polymer in the swollen sample (υ) are calculated as follows:

(1) $$w\left(\%\right)=\frac{\left(m_{\text{unex}}-m_{\text{ex}}\right)}{m_{\text{unex}}}\times 100$$

(2) $$\upsilon ={\left[1+\frac{\left(m_{s}-m_{\text{ex}}\right)}{m_{\text{ex}}}\times \frac{\rho_{p}}{\rho_{s}}\right]}^{-1}$$

where ρ_s_ and ρ_p_ are the solvent (toluene, 0.865 g/cm^3^) and polymer (PDMS, 0.965 g/cm^3^) densities, respectively. Swelling experiments were carried out five times for each test.

IR data of the cross-linking, structure-activity relationships of *trans*-(**1**–**4**), stability of *cis*-**4** in polysiloxane solution in air, DSC fata for silicone rubbers, and TG measurements are given in Supplementary Materials.

## 4. Conclusions

The results of this work could be considered from at least three perspectives. Firstly, in the field of catalysis, we observed that isomeric platinum(II) complexes featuring benzyl- and phenylcyanides and also dialkylcyanamides, *cis*- and *trans*-(**1**–**4**), behave as efficient catalysts for the cross-linking of vinyl terminated polydimethylsiloxane and trimethylsilyl terminated poly(dimethylsiloxane-*co*-ethylhydrosiloxane). Among the tested platinum species, *cis*-complexes are much more active catalysts than their *trans*-congeners and, for all tested complexes, *cis*-[PtCl_2_(NCCH_2_Ph)_2_] (*cis*-**4**) exhibit the best catalytic activity.

Secondly, although *cis*-**4** is less active than the widely used Karstedt’s catalyst, its application for the cross-linking can be performed not only at RT (*c* = 1.0 × 10^−4^ mol/L), but also, more efficiently, at 80 °C (*c* = 1.0 × 10^−4^−1.0 × 10^−5^ mol/L), which is crucial for some industrial applications. The cross-linking involving the nitrile Pt^II^ complexes as the catalysts can be conducted without inhibitors and, moreover, the nitrile platinum species and their mixtures with vinyl- and trimethylsilyl terminated polysiloxanes are shelf-stable in air and all these properties provide further benefits. In addition, our catalysts visually do not form colloid platinum particles after the cross-linking.

Recently, we reported that shelf-stable and moisture/air insensitive platinum(II) imino complexes—obtained by a click-type coupling between a hydroxyguanidine and (nitrile)Pt^II^ species—catalyze hydrosilylation cross-linking of vinyl terminated poly(dimethylsiloxane) and trimethylsilyl terminated poly(dimethylsiloxane-co-ethylhydrosiloxane) giving (in air and at 50 °C) high quality thermally stable silicon resins [30]. Comparison of the previous [30] and current catalytic systems indicate that the latter suggest more benefits. Indeed, in contrast to the previously reported catalysts, the current catalytic system (in particular, *cis*-**4**) operates at a substantially wider temperature range (25–80 °C) and the obtained silicone formulations are more thermally stable (by *ca.* 30 °C) and their dynamic characteristics are substantially better, *viz.* E′ is six times higher.

Thirdly, in the area of silicon polymer chemistry, in contrast to rubbers obtained with Karstedt’s catalyst, PDMS-EHDMS silicone formulations obtained with any one of *cis*-(**1**–**4**) bear no bubbles or other structural defects, and their surface is homogeneous and transparent. These rubbers can be stored in the light and in air for at least one year without visible changes. The application of *cis*-**4** for the cross-linking largely improves thermal stability and flame (fire) retardancy of the silicone rubber compared with those generated with Karstedt’s catalyst. Our data indicate the formation of networks with significantly fewer defects (*i.e.*, unreacted vinyl and hydride groups) and higher cross-linking density in the presence of the nitrile platinum species unlike Karstedt’s catalyst.

## Figures and Tables

**Table 1:** Parameters of the catalytic cross-linking of PDMS and EHDMS.

Catalyst	с_catalyst_, mol/L	τ_pot-life_ at RT, min	τ_curing_ at RT	τ_curing_, min at 80 °С	DSC Data			−ΔH, J/g
					T_onset_, °С	T_peak_, °С	T_endset_, °С	
*cis*-**1**	1.0 × 10^−3^	120	24 h	20	87.2	105.8	121.6	8.6
*cis*-**2**	1.0 × 10^−3^	150	24 h	10	82.2	101.8	118.0	10.1
*cis*-**3**	1.0 × 10^−3^	120	20 h	10	84.6	92.1	118.0	15.5
*cis*-**4**	1.0 × 10^−3^	20	3 h	5	not measured			
	1.0 × 10^−4^	60	6 h	30	84.7	90.6	94.2	17.2
	5.0 × 10^−5^	260	36 h	60	83.8	106.6	111.5	17.2
	1.0 × 10^−5^	−	30 day	600	115.2	145.4	153.3	24.1
*trans*-**1**	1.0 × 10^−3^	−	30 day	120	133.0	141.6	145.0	10.4
*trans*-**2**	1.0 × 10^−3^	−	14 day	80	126.9	130.3	133.4	10.7
*trans*-**3**	1.0 × 10^−3^	−	5 day	60	110.2	115.3	120.2	10.1
*trans*-**4**	1.0 × 10^−3^	240	24 h	15	98.6	108.8	115.6	7.6
	1.0 × 10^−5^	−	20 day	80	107.6	133.0	157.5	19.0
Karstedt’s catalyst	1.0 × 10^−4^	0.7	0.1 h	−	not measured			
	5.0 × 10^−5^	1.1	0.2 h	−	not measured			
	1.0 × 10^−5^	3	6 h	−	35.4	51.3	65.9	4.5

## Supplementary Materials

Supplementary materials can be accessed at: http://www.mdpi.com/1420-3049/21/3/311/s1.
