# Supplementary material for: bis-Nitrile and bis-Dialkylcyanamide Platinum(II) Complexes as Efficient Catalysts for Hydrosilylation Cross-Linking of Siloxane Polymers
Source: Molecules. 2016 Mar 5;21(3):311. doi: 10.3390/molecules21030311 (PMC6274487; doi:10.3390/molecules21030311)
Supplement: Supplementary file 1 [file molecules-21-00311-s001.pdf]

Supplementary Materials: *bis*-Nitrile and *bis*-Dialkylcyanamide Platinum(II) Complexes as Efficient Catalysts for Hydrosilylation Cross-linking of Siloxane Polymers

Regina M. Islamova, Mikhail V. Dobrynin, Daniil M. Ivanov, Andreii V. Vlasov, Elena V. Kaganova, Galina V. Grigoryan and Vadim Yu. Kukushkin

IR Data of the Cross-linking

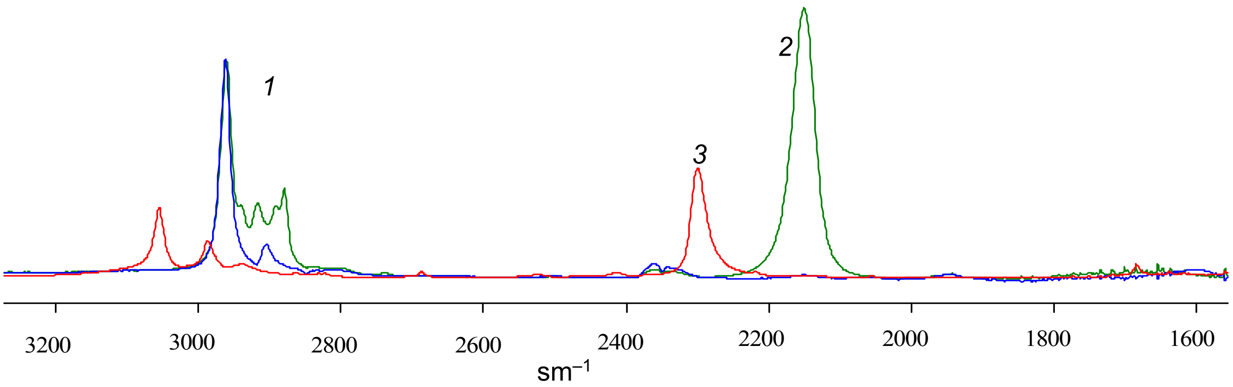

Figure S1. The IR spectra of PDMS (1), EHDMS (2), and *cis*-**1** (3) (1.0×10^{-3} mol/L).

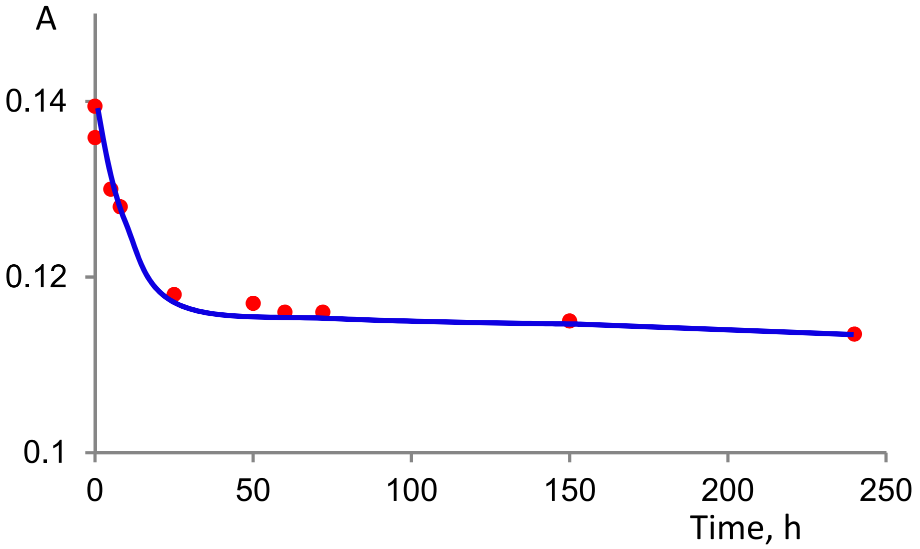

Figure S2. Intensity of absorption band at 2152 cm^{-1} *vs.* time of the cross-linking with *cis*-**1** (1.0×10^{-3} mol/L).

Structure-Activity Relationships of *trans*-(1–4)

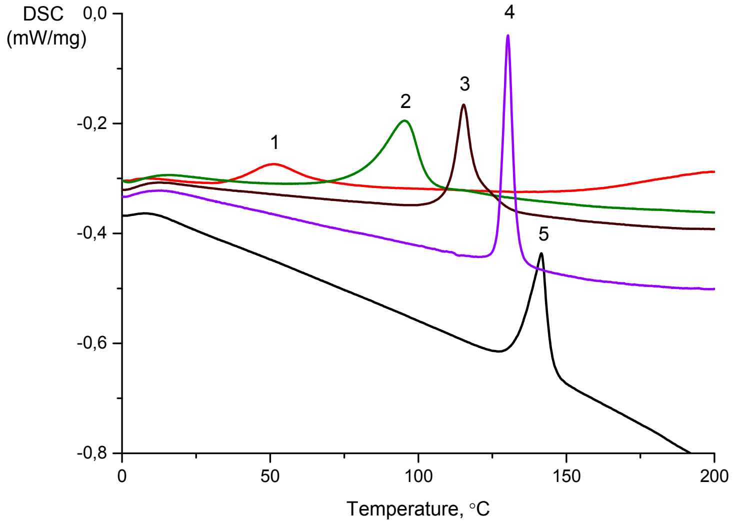

Figure S3. DSC curves of the curing catalyzed by: Karstedt's catalyst (1.0×10^{-5} mol/L) (1); *trans*-**4** (1.0×10^{-3} mol/L) (2); *trans*-**3** (1.0×10^{-3} mol/L) (3); *trans*-**2** (1.0×10^{-3} mol/L) (4); *trans*-**1** (1.0×10^{-3} mol/L) (5).

Stability of *cis*-4 in Polysiloxane Solution in Air

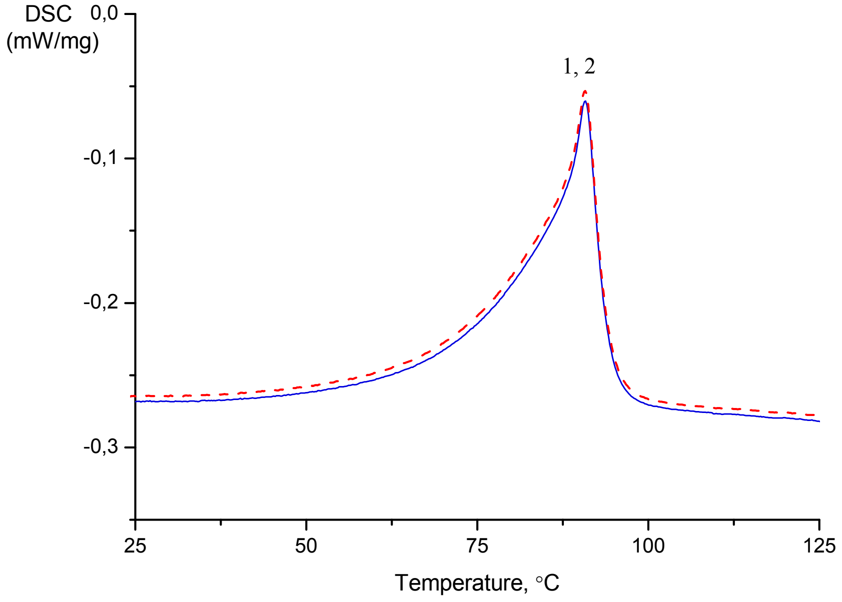

Figure S4. DSC curves of the PDMS and EHDMS cross-linking catalyzed by *cis*-**4** (1.0×10^{-4} mol/L) immediately after mixing (1) and by *cis*-**4** (1.0×10^{-4} mol/L) after 30 days (2).

DSC Data for Silicone Rubbers

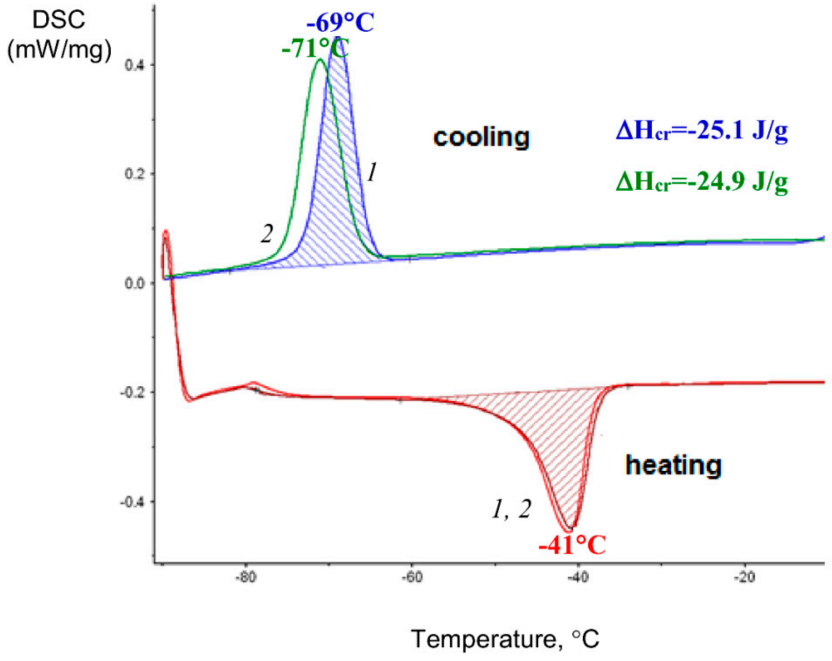

Figure S5. Effect of the nature of platinum catalyst on the crystallization and melting of PDMS–EHDMS silicon rubber: 1–Karstedt's catalyst, 2–**1** (1.0×10^{-3} mol/L).

TG Measurements

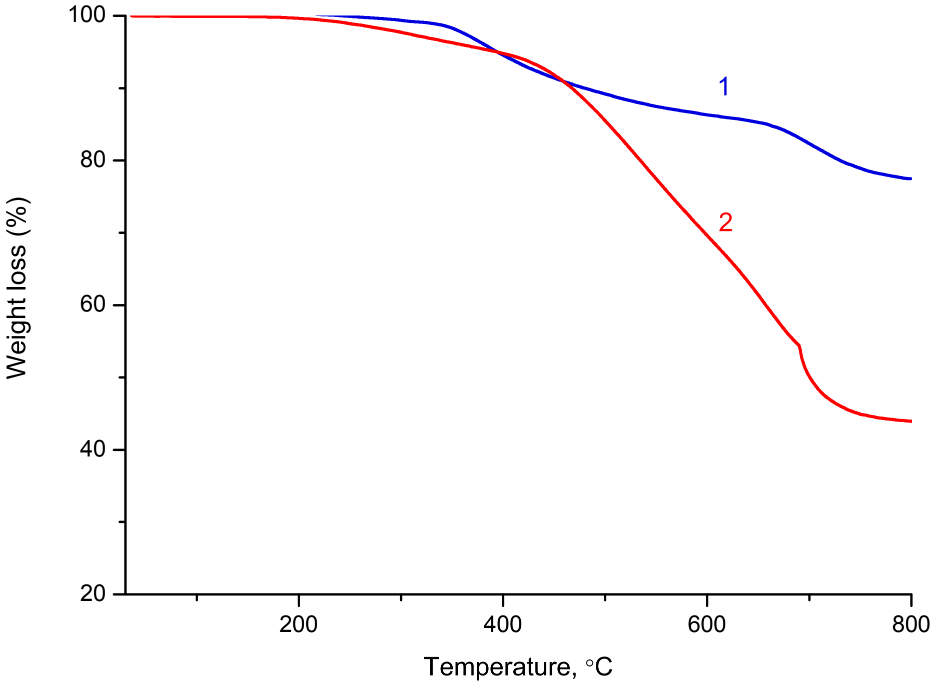

Figure S6. Effect of the nature of platinum catalyst on the thermal degradation of the PDMS–EHDMS silicon rubber in argon. 1–*cis*-**4** (1.0×10^{-5} mol/L); 2–Karstedt's catalyst (1.0×10^{-5} mol/L).

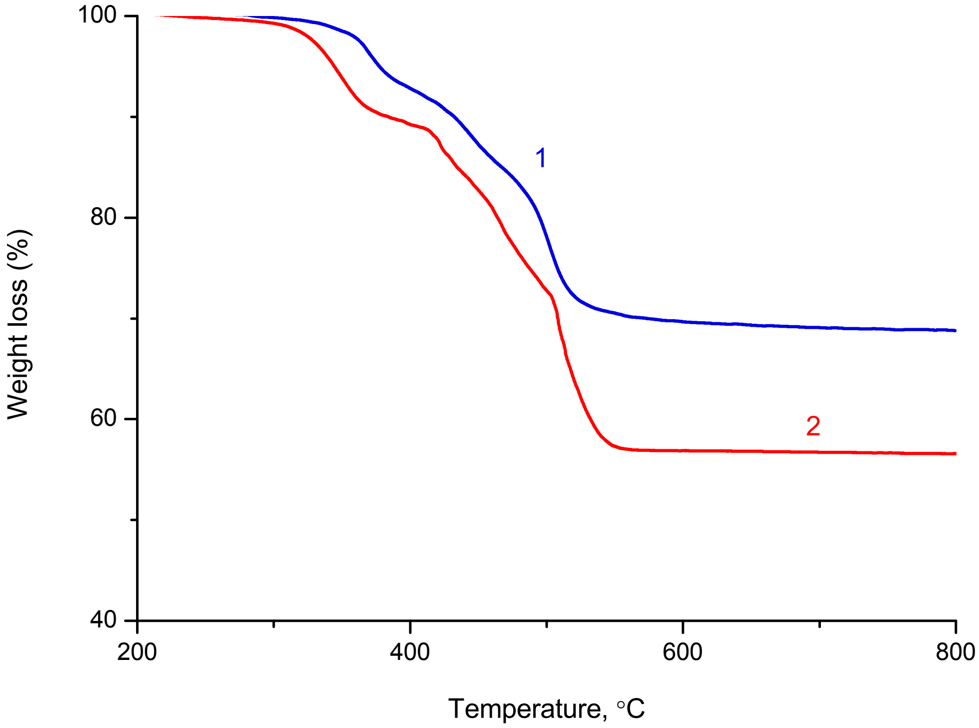

Figure S7. Effect of the nature of platinum catalyst on the thermal degradation of the PDMS–EHDMS silicon rubber in air. 1–*cis*-**1** (1.0×10^{-5} mol/L); 2–Karstedt's catalyst (1.0×10^{-5} mol/L).
